# Quantifying sodium [^18^F]fluoride uptake in abdominal aortic aneurysms

**DOI:** 10.1186/s13550-022-00904-z

**Published:** 2022-06-06

**Authors:** Samuel Debono, Jennifer Nash, Alexander J. Fletcher, Maaz B. J. Syed, Scott I. Semple, Edwin J. R. van Beek, Alison Fletcher, Sebastien Cadet, Michelle C. Williams, Damini Dey, Piotr J. Slomka, Rachael O. Forsythe, Marc R. Dweck, David E. Newby

**Affiliations:** 1 The University of Edinburgh Centre for Cardiovascular Science, Chancellor’s Building, University of Edinburgh, 49 Little France Crescent, Edinburgh, EH16 4SB UK; 2 Edinburgh Imaging Facility, Queen’s Medical Research Institute, University of Edinburgh, Edinburgh, UK; 3 Division of Artificial Intelligence, Department of Medicine, Cedars-Sinai Medical Centre, Biomedical Imaging Research Institute, Los Angeles, USA

**Keywords:** Aneurysms, Positron emission tomography, Image analysis, Radiotracer

## Abstract

**Background:**

Aortic microcalcification activity is a recently described method of measuring aortic sodium [^18^F]fluoride uptake in the thoracic aorta on positron emission tomography. In this study, we aimed to compare and to modify this method for use within the infrarenal aorta of patients with abdominal aortic aneurysms.

**Methods:**

Twenty-five patients with abdominal aortic aneurysms underwent an sodium [^18^F]fluoride positron emission tomography and computed tomography scan. Maximum and mean tissue-to-background ratios (TBR) and abdominal aortic microcalcification activity were determined following application of a thresholding and variable radius method to correct for vertebral sodium [^18^F]fluoride signal spill-over and the nonlinear changes in aortic diameter, respectively. Agreement between the methods, and repeatability of these approaches were assessed.

**Results:**

The aortic microcalcification activity method was much quicker to perform than the TBR method (14 versus 40 min, *p* < 0.001). There was moderate-to-good agreement between TBR and aortic microcalcification activity measurements for maximum (interclass correlation co-efficient, 0.67) and mean (interclass correlation co-efficient, 0.88) values. These correlations sequentially improved with the application of thresholding (intraclass correlation coefficient 0.93, 95% confidence interval 0.89–0.95) and variable diameter (intraclass correlation coefficient 0.97, 95% confidence interval 0.94–0.99) techniques. The optimised method had good intra-observer (mean 1.57 ± 0.42, bias 0.08, co-efficient of repeatability 0.36 and limits of agreement − 0.43 to 0.43) and inter-observer (mean 1.57 ± 0.42, bias 0.08, co-efficient of repeatability 0.47 and limits of agreement − 0.53 to 0.53) repeatability.

**Conclusions:**

Aortic microcalcification activity is a quick and simple method which demonstrates good intra-observer and inter-observer repeatabilities and provides measures of sodium [^18^F]fluoride uptake that are comparable to established methods.

**Supplementary Information:**

The online version contains supplementary material available at 10.1186/s13550-022-00904-z.

## Introduction

Sodium [^18^F]fluoride positron emission tomography and computed tomography (PET-CT) is a non-invasive multimodality imaging technique that detects early calcification activity as a marker of vascular injury [1]. Conventional CT imaging can visualise established larger macrocalcified plaques. Sodium [^18^F]fluoride binds to microscopic hydroxyapatite and pyrophosphate crystals to identify earlier microcalcification on PET which is beyond the resolution of CT [2, 3]. Sodium [^18^F]fluoride PET has thus emerged as a promising imaging biomarker for the early detection of vascular injury and calcification activity [4].

Abdominal aortic aneurysms are degenerative aortic conditions characterised by widespread cellular destruction and loss of medial architecture. It has been previously shown that aortic sodium [^18^F]fluoride PET uptake is higher within aneurysmal aortic segments compared to non-aneurysmal segments and matched control subjects without aortopathy [5]. In addition, abdominal aortic aneurysms with the highest sodium [^18^F]fluoride uptake experience the greatest rates of aneurysm growth and are associated with a greater likelihood of aortic rupture or elective repair [5]. This relationship is independent of CT calcium score and the maximum aneurysm diameter, the current gold standard to predict major adverse aortic events. The accurate detection of sodium [^18^F]fluoride binding in patients with abdominal aortic aneurysms thus holds great promise to improve risk stratification and potentially guide interventions.

Aortic microcalcification activity (AMA) is a recently described simplified method of measuring aortic sodium [^18^F]fluoride uptake [6]. It is quick to perform and correlates well with clinical outcomes. However, this technique has only been applied in the thoracic aorta. This study’s aim was to assess the AMA method for quantifying sodium [^18^F]fluoride uptake within the infrarenal aorta of patients with abdominal aortic aneurysms by comparing it with the established method of tissue-to-background ratio. Specifically, (1) to investigate the comparability of these measurements, (2) to assess modifications to account for spill-over of the sodium [^18^F]fluoride signal from adjacent vertebra and the variable aneurysm diameter, and (3) to determine the within and between observer repeatability of the optimised analytical approach [7].

## Methods

### Study population

The study population comprised 25 consecutive patients recruited into the sodium [^18^F]fluoride Imaging in Abdominal Aortic Aneurysms study (NCT02229006). Participants were aged over 50 years and under routine clinical surveillance with an asymptomatic abdominal aortic aneurysm defined as ≥ 40 mm inner-to-inner anteroposterior diameter on ultrasound.

### Sodium [18F]fluoride PET-CT

Patients were administered a target dose of 125 MBq of sodium [^18^F]fluoride intravenously and after 60 min were imaged on a hybrid 128–slice PET-CT scanner (Biograph mCT, Siemens Healthineers, Erlangen, Germany) [8]. A low-dose attenuation correction CT scan was performed (120 kV, 50 mAs, 5/3 mm), followed by acquisition of PET data at 10-min intervals in three bed positions to ensure complete coverage of the thoracic and abdominal aorta. Contrast-enhanced CT angiography (120 kV, 145 mAs, 3/3 mm, field of view 400; and 1/1 mm, field of view 300; triggered at 181 Hounsfield units) was performed on the same scanner immediately after PET acquisition. This was centred on the abdominal aortic aneurysm and extended to the aortic bifurcation.

Static PET-CT images were reconstructed with correction applied for attenuation, deadtime, scatter and random coincidences, using an optimised iterative reconstruction algorithm (ultra-High Definition; TrueX + Time-of-Flight, 2 iterations and 21 subsets, matrix 200, zoom 1; Gaussian filter 5 mm).

### Image analysis

A custom validated tool was used to quantify sodium [^18^F]fluoride uptake (Fusion Quant v1.21.0421, Cedars-Sinai Medical Centre, Los Angeles) [9].

#### Background blood pool

The background blood pool activity was determined by placing two 8-mm radius spheres in the centre of the right and left atria. The cumulative standard uptake values (SUVs) within the spheres was then corrected for the spheres’ total volume (2.1 cm^3^). The mean background pool activity was then used in tissue-to-background ratio and aortic microcalcification activity calculations as well as a minimum visualisation threshold.

#### Volumes of interest within the aorta

**Fig. 1:**
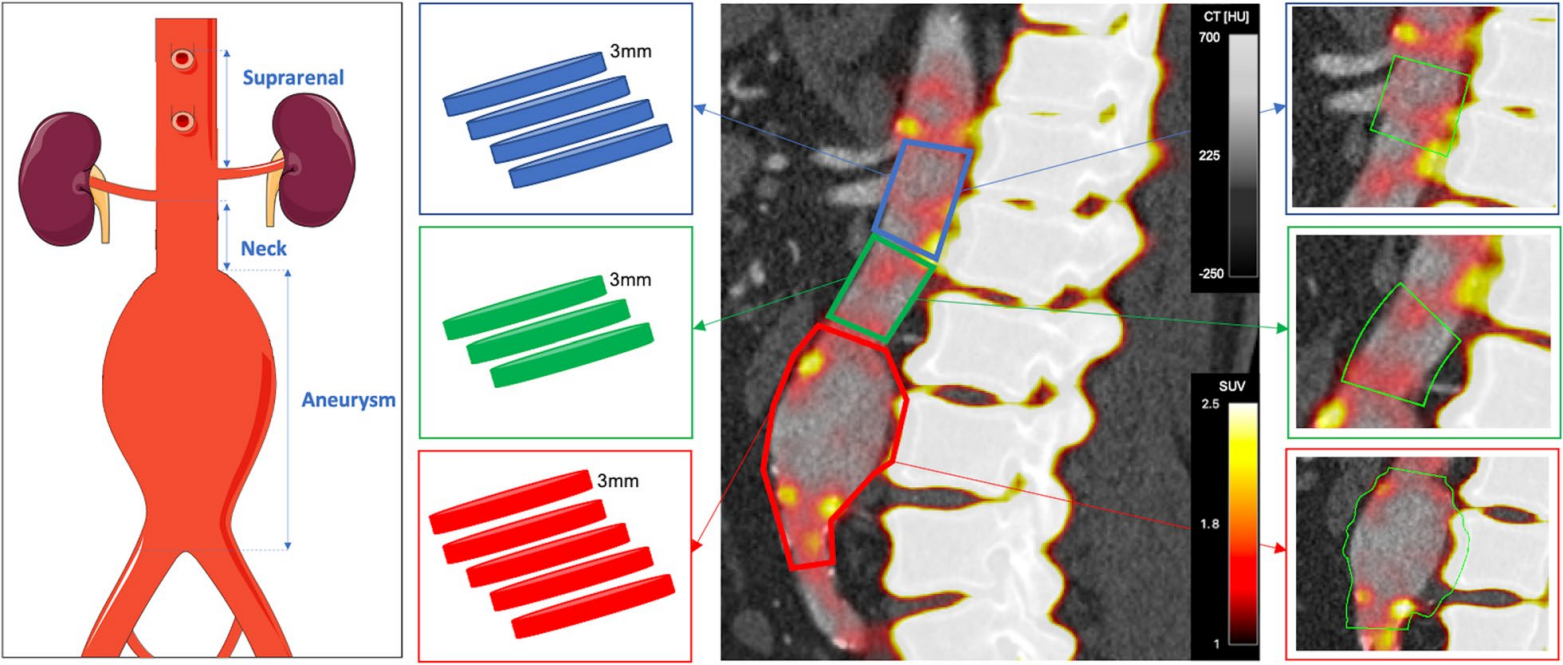
Regions of interest within the abdominal aorta. The abdominal aorta was split into three anatomical regions for analysis demonstrated on the panel on the left. Suprarenal—starting from the level of the origin of the coeliac artery till the upper-most renal artery. Neck—starting from the lower-most renal artery until where the aorta becomes aneurysmal. Aneurysm—starting just after the neck region ends until the aortic bifurcation. The second panel from the left shows a graphic representation of sequential 3 mm polygons taken from the three different aortic regions (suprarenal—blue, neck—green, aneurysm—red). The third panel from the left shows a sagittal view of the abdominal aorta. The right panel shows the volumes of interest drawn on the image analysis programme. CT [HU], computed tomography grey scale bar in Hounsfield units; SUV, positron emission tomography colour scale bar in standard uptake values

On the attenuation correction CT, the thoracic aorta was defined as the region where the first trans-axial slice of the descending aorta starts until the aortic hiatus at the diaphragm [10]. Being of normal diameter and non-aneurysmal, the thoracic aorta was considered as a control. Using the CT angiogram, the abdominal aorta was then analysed in three separate sections (Fig. 1): (1) the ‘suprarenal aorta’ was defined as the origin of the coeliac artery down to the origin of the upper most renal artery; (2) the ‘neck’ was defined as the origin of the lower most renal artery until the abdominal aorta became aneurysmal, or there was a definite change in vessel calibre (the latter applied to cases where the neck was ectatic); and (3) the aneurysm sac was defined as where the neck ended until the aortic bifurcation.

#### Tissue-to-background ratio

Regions of interest with a thickness of 3 mm were drawn around the aorta in the trans-axial plane along the entire length of the thoracic aorta and each aortic segment [10]. For each region, mean and maximum SUVs (SUV_mean_ and SUV_max_, respectively) were measured (Fig. 1). These values were then divided by the background pool activity to obtain tissue-to-background ratios (TBRs) for both the mean (TBR_mean_) and maximum (TBR_max_) values. Care was taken to exclude regions of overspill from vertebral sodium [^18^F]fluoride uptake.

#### Abdominal aortic microcalcification activity

The image analysis programme has a centreline function in a multiplanar reconstruction viewer. This allows the creation of a volume of interest with an adjustable radius and length along a centreline which can be adjusted for the vessels’ shape and angulation as necessary. The volume of interest included the aortic lumen, any thrombus present and the aortic wall. The diameter for each volume of interest matched that of the maximal aortic luminal diameter at that point in the centreline. Four different centrelines were drawn for each aortic segment: thoracic, suprarenal, neck and body of the aneurysm (Fig. 1). The cumulative SUV (mean) of each volume of interest created is thus obtained, along with its volume and maximum SUV. Similarly to the previously described AMA method [6], the cumulative SUV for each of the aortic segments was then divided by the volume and the mean background pool activity to obtain the mean AMA value.

The method was however further enhanced for the abdominal aorta in two ways:

1. *Maximum Threshold* Sodium [^18^F]fluoride is physiologically taken up by the vertebrae. This creates a spill-over effect where there is spill-over of the radiotracer signal into the surrounding tissues including the aneurysm. For each aortic region, a separate 3-D sphere was drawn in the visually highest uptake area, this had to be clearly distinct from the vertebra. The SUV_max_ within this sphere was then applied as an upper voxel intensity threshold for the corresponding region’s volume of interest. Any values above this SUV were automatically excluded in the cumulative SUV and volume for that region by the analysis programme. The values for each region were obtained twice, once with the threshold limit applied and once without.
2. *Aneurysm variable radius* Using a uniform centreline function (3-D cylinder) is sufficient if the volume of interest is of the same diameter throughout. Within an abdominal aortic aneurysm, the diameter varies along its length and if the centreline shape is kept uniform, this would lead to inclusion of extra-aortic tissue or exclusion of aneurysm tissue. A varying radius function was therefore introduced to allow the radius of the centreline to be varied across different points of the centreline to capture the aneurysmal volume of interest more accurately. The aneurysm values were obtained twice, with and without a variable radius.

The maximum AMA (AMA_max_) was also calculated by dividing the SUV_max_ by the background pool activity.

### Intra-observer and inter-observer repeatabilities

The AMA method was repeated for all 25 patients by two trained observers (SD, JN). To minimise recall bias, intra-observer repeatability was assessed by the same trained researcher (SD) using repeated assessments performed 3 months apart in random order. Duration of analyses were recorded for each method of assessment.

### Statistical analysis

Statistical analysis was performed using statistical software package R (v4.0.2, R Foundation for Statistical Computing, Vienna). Continuous variables with normal distribution were presented as mean ± standard deviation, whereas skewed continuous variables were presented as median [interquartile range]. Categorical variables were presented as number (percentage). Associations between quantification methods were evaluated as a continuous variable (Pearson’s correlation coefficient). Quantification methods were compared using intraclass correlation coefficient (consistency and 2-way random effects model) [11] and Bland–Altman plots [12]. Reliability of intraclass correlation coefficient values was described as: poor when less than 0.5; moderate when 0.5–0.75; good when 0.75–0.9; and excellent when greater than 0.9 [11]. Intra- and inter-observer repeatabilities were similarly assessed using mean bias, 95% limits of agreement and coefficient of repeatability [13]. Statistical significance was taken as a two-sided *p* < 0.05.

## Results

**Fig. 2:**
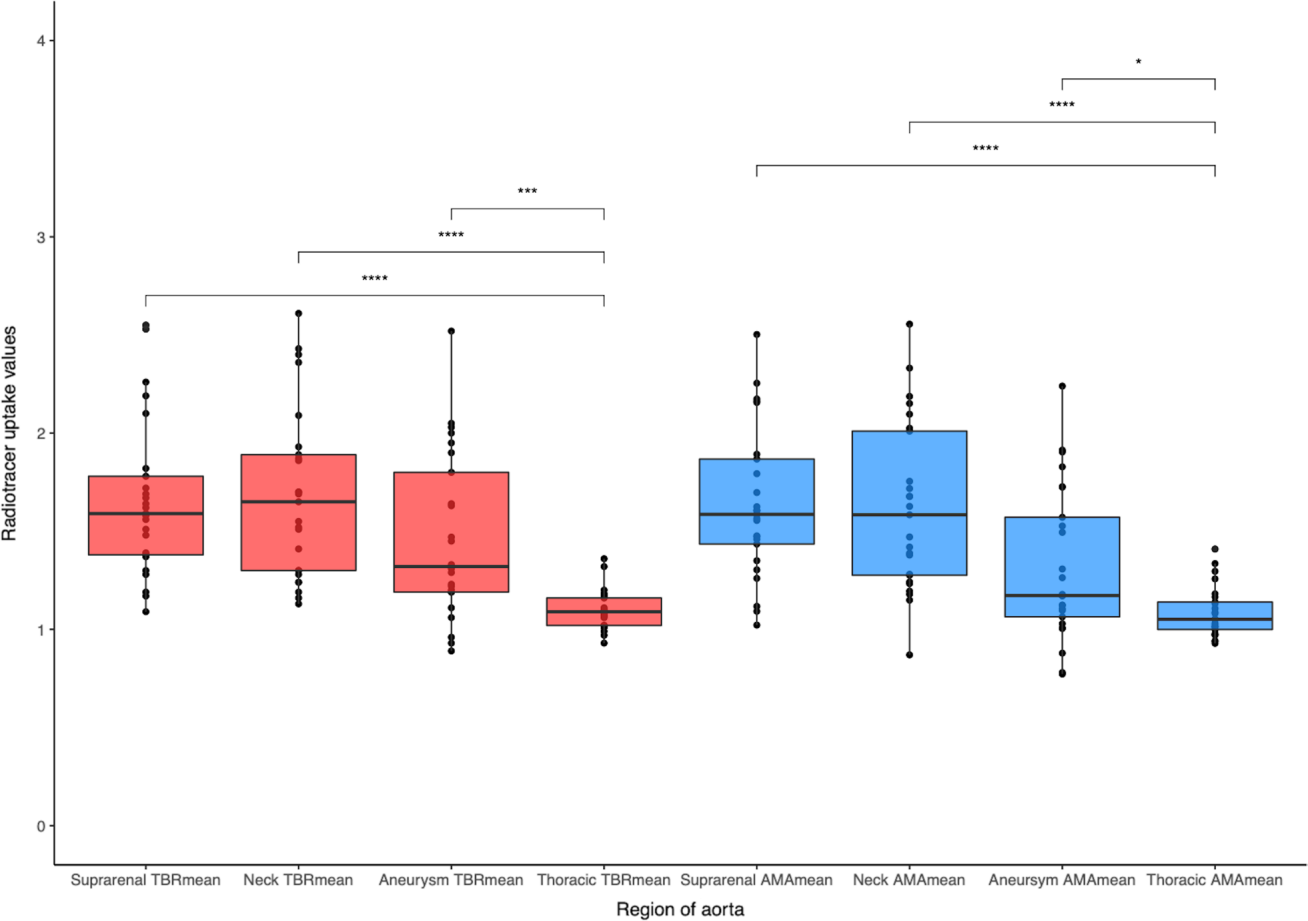
Radiotracer uptake in different regions of the aorta. Mean tissue-to-background ratio (red) and aortic microcalcification activity (blue) in the regions of the abdominal aorta and the thoracic aorta. AMA, aortic microcalcification activity; TBR, tissue-to-background ratio. * = *p* < 0.05, *** = *p* < 0.001, **** = *p* < 0.0001

Patients had a median age of 72 years and were predominantly male (Table 1). Sodium [^18^F]fluoride uptake was present in the thoracic and abdominal aorta of all 25 patients, although it varied between the thoracic aorta and the three regions of the abdominal aorta for both the TBR and AMA methods (Fig. 2 and Additional file1: Fig. S1). The AMA method was quicker to perform, with the TBR method taking approximately 26 min longer (14 [13–17] versus 40 [34–44] min, *p* < 0.001).

**Table 1:** Patient characteristics

Characteristic	*N* = 25
Age (years)	72 [61–83]
Male	21 (84%)
Female	4 (16%)
Systolic blood pressure (mmHg)	138 [101–180]
Diastolic blood pressure (mmHg)	81 [56–112]
Heart rate (beats/min)	72 [58 to 86]
Body mass index (kg/m^2^)	27.0 [20.2–36.3]
*Medical history*	
Current smoker	8 (33%)
Hypertension	18 (72%)
Hypercholesterolaemia	21 (84%)
Diabetes	5 (20%)
Ischaemic heart disease	6 (24%)
Peripheral arterial disease	6 (24%)
Cerebrovascular disease	3 (12%)
Family history of aneurysms	4 (16%)
*Medication*	
Antiplatelet agents	17 (68%)
Statins	21 (84%)
Anticoagulant agents	2 (8.0%)
Beta-blockers	7 (28%)
Angiotensin-converting enzyme inhibitors or Angiotensin receptor blockers	13 (52%)
*Aorta*	
Aortic diameter (mm)	46 [40–85]
Concurrent iliac aneurysm	6 (24%)
Subsequent aortic repair	5 (20%)

Characteristics of the twenty study patients including their medical history, current medication and aortic features. Median [Range]; number (%)

### Abdominal aortic aneurysm sodium [18F]fluoride uptake

#### Maximum values

**Fig. 3:**
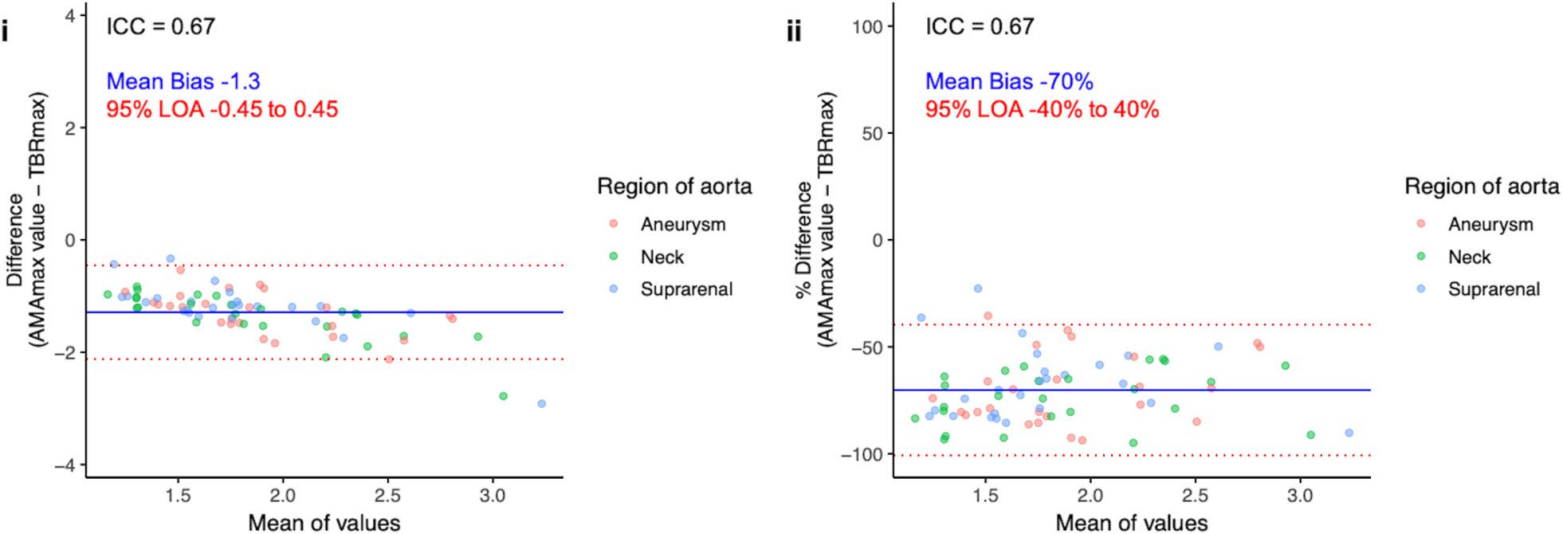
Maximum tissue-to-background ratio (TBR_max_) and maximum abdominal Aortic Microcalcification Activity (AMA_max_). Bland–Altmann plots with actual difference (left), and percentage difference (right) with mean bias (blue line) and 95% limits of agreement (red lines) for TBR_max_ and AMA_max_. Y-axis limits are set to the mean of the values + 2 and − 2 and + 1% and − 1%, respectively. AMA, aortic microcalcification activity; ICC, intraclass correlation coefficient; LOA, limits of agreement; max, maximum; TBR, tissue-to-background ratio

Within the abdominal aorta, TBR_max_ values ranged from 1.41 to 4.69 with a mean of 2.49 ± 0.65, and AMA_max_ values ranged from 0.68 to 2.12 with a mean of 1.2 ± 0.35. While the values were correlated (*r* = 0.79, *p* < 0.001; Additional file1: Fig. S2), there was evidence of substantial bias and wide limits of agreement when comparing the two approaches (Fig. 3). Overall, there was moderate agreement between TBR_max_ and AMA_max_ (intraclass correlation coefficient 0.67, 95% confidence interval 0.52–0.78).

#### Mean values

**Fig. 4:**
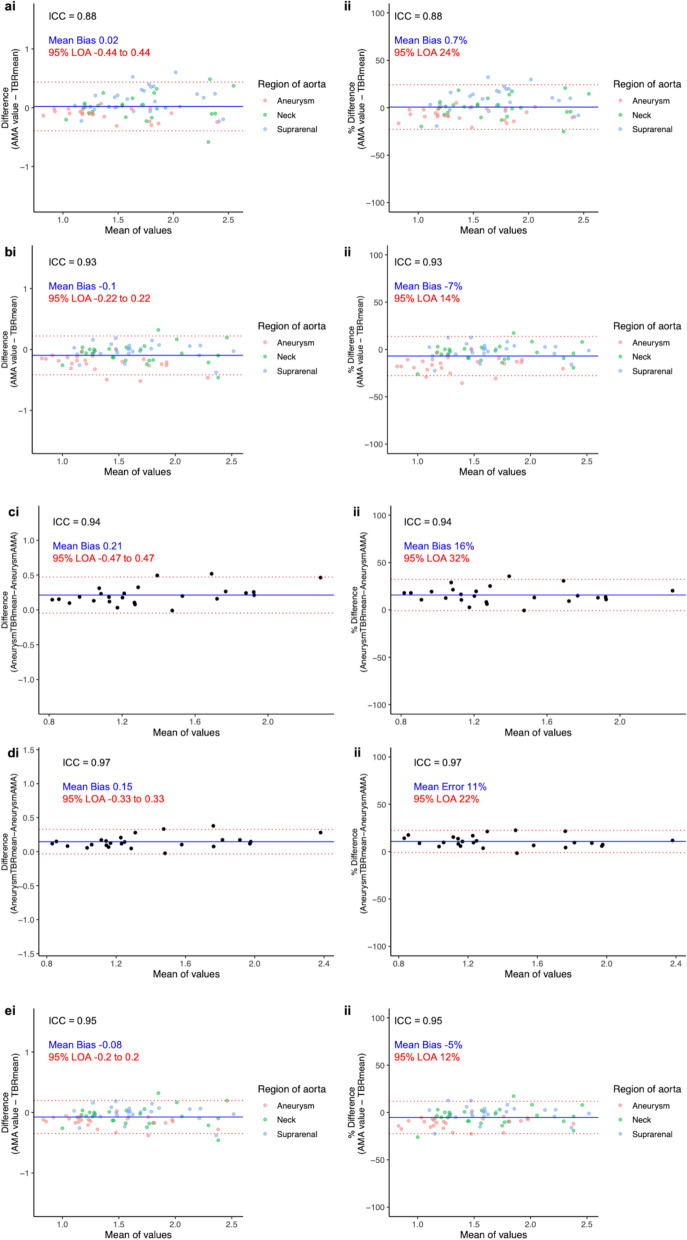
Mean tissue-to-background ratio (TBR_mean_) and mean abdominal Aortic Microcalcification Activity (AMA). Bland-Altmann plots with actual difference (left side) and percentage difference (right side), mean bias (blue line) and 95% limits of agreement (red lines) for: All regions without applying threshold (**a**), all regions after applying threshold (**b**), aneurysm region without variable radius (**c**), aneurysm region with variable radius (**d**), all regions after applying both threshold and variable radius (**e**). Y-axis limits in the actual difference plots are set to the mean of the values. AMA, aortic microcalcification activity; ICC, intraclass correlation coefficient; LOA, limits of agreement; TBR, tissue-to-background ratio

TBR_mean_ values ranged from 0.89 to 2.61 with a mean of 1.6 ± 0.42, and AMA_mean_ values ranged from 0.75 to 2.73 with a mean of 1.62 ± 0.44. The values were highly correlated (*r* = 0.95, *p* < 0.001; Additional file1: Fig. S2) with lower bias and narrower limits of agreement (Fig. 4) as well as very good agreement (Table 2).

**Table 2:** Comparison of mean aortic microcalcification activity to mean tissue-to-background ratio

	Range	Mean	Mean Bias (95% limits of agreement)	Intraclass Correlation Coefficient
AMA without threshold	0.75–2.73	1.62 ± 0.44	0.02 (− 0.44 to 0.44)	0.88
AMA with threshold	0.74–2.56	1.5 ± 0.43	− 0.1 (− 0.22 to 0.22)	0.93
Aneurysm AMA without variable radius	0.74–2.06	1.25 ± 0.36	0.21 (− 0.47 to 0.47)	0.94
Aneurysm AMA with variable radius	0.77–2.24	1.32 ± 0.39	0.15 (− 0.33 to 0.33)	0.97
AMA	0.77–2.56	1.53 ± 0.42	− 0.08 (− 0.19 to 0.19)	0.95

Comparison of mean aortic microcalcification activity to mean tissue-to-background ratio detailing the mean bias and intraclass correlation coefficient between the different levels of enhancement. AMA, aortic microcalcification activity; Mean ± standard deviation

### Enhanced image analysis technique

#### Maximum threshold

After applying the maximum threshold technique, there was good to excellent agreement between TBR_mean_ and AMA_mean_ (intraclass correlation coefficient 0.93, 95% confidence interval 0.89–0.95). Similarly, there were marked improvements in the mean bias and 95% limits of agreement (Table 2, Fig. 4).

#### Aneurysm variable radius

There was good to excellent agreement between TBR_mean_ and AMA_mean_ without the variable radius approach (intraclass correlation coefficient 0.94, 95% confidence interval 0.88–0.98). This was further improved with the application of a variable radius (intraclass correlation coefficient 0.97, 95% confidence interval 0.94–0.99). This approach was also associated with improvements in bias and limits of agreement between the two measures (Table 2, Fig. 4). Finally, applying both these techniques resulted in excellent agreement between TBR_mean_ and AMA_mean_ (Table 2, Fig. 4).

### Intra-observer and inter-observer repeatabilities

**Fig. 5:**
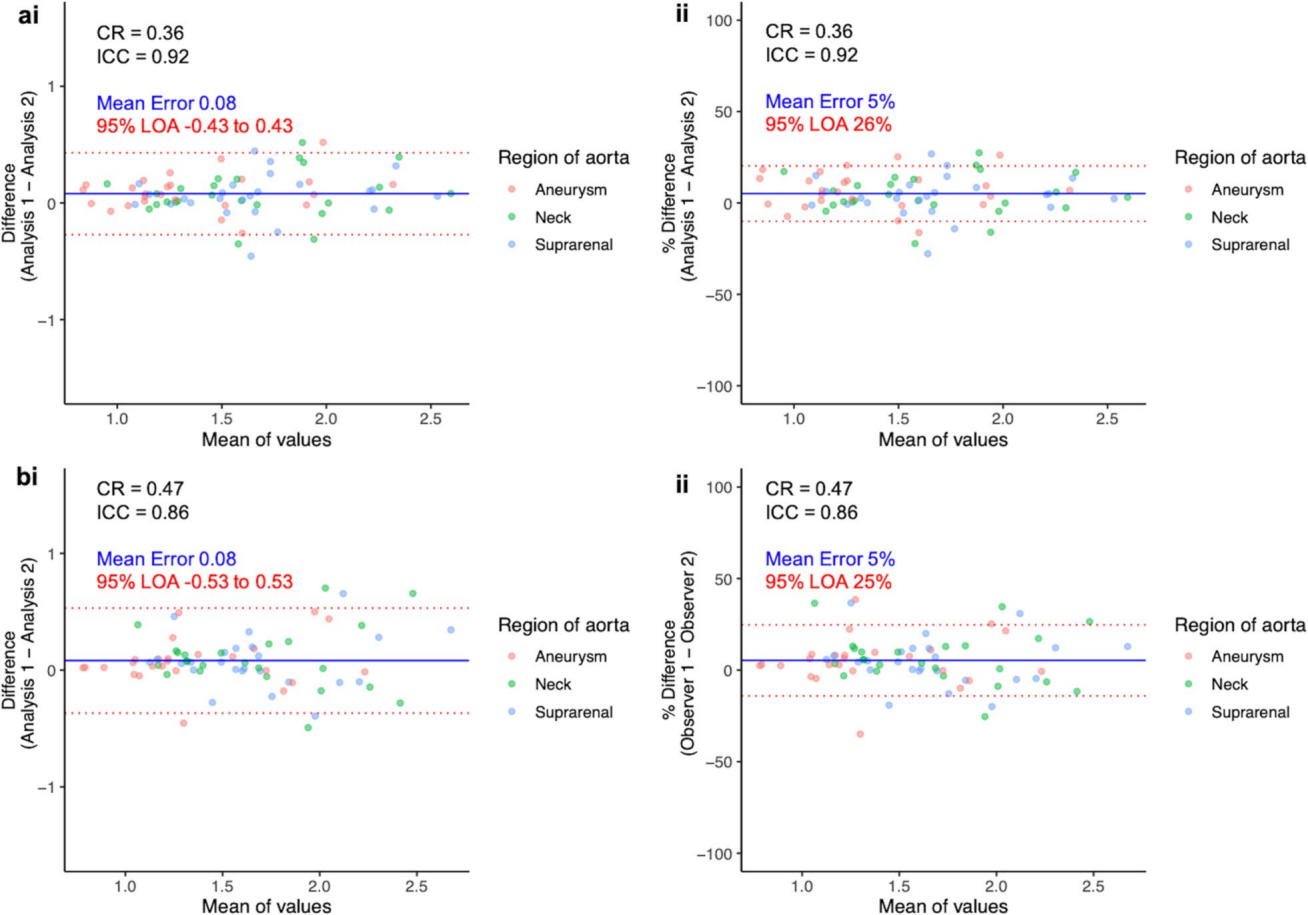
Intra-observer and inter-observer repeatability. Bland-Altmann plots with actual difference (left side) and percentage difference (right side), mean error (blue line) and 95% limits of agreement (red lines) for Intra-observer values (**a**), and inter-observer (**b**). Y-axis limits in the actual difference plots are set to the mean AMA value. AMA, aortic microcalcification activity; CR, coefficient of reproducibility; ICC, intraclass correlation coefficient; LOA, limits of agreement; TBR, tissue-to-background ratio

Intra-observer and inter-observer assessments were highly correlated (Additional file1: Fig. S2) and demonstrated good to excellent repeatability (Table 3, Fig. 5).

**Table 3:** Intra-observer and inter-observer repeatabilities

	Range	Mean	Mean bias (95% limits of agreement)	Coefficient of repeatability (% of mean)	Intraclass correlation Coefficient
Intra-observer	0.77–2.64	1.57 ± 0.42	0.08 (− 0.43 to 0.43)	0.36 (23.0)	0.92
Inter-observer	0.77–2.85	1.57 ± 0.42	0.08 (− 0.53 to 0.53)	0.47 (30.0)	0.86

Mean bias, coefficient of repeatability and intraclass correlation coefficient of intra-observer and inter-observer values. Mean ± standard deviation

## Discussion

This is the first description of applying the AMA method to the abdominal aorta. This method has excellent levels of agreement and is substantially quicker than previously described conventional PET quantification methods. Moreover, it performs much better when incorporating modifications that account for the spill-over of sodium [^18^F]fluoride uptake from the adjacent vertebrae and the variable aortic radius of the aneurysm. This quick and highly repeatable technique will improve the practical application and analysis of sodium [^18^F]fluoride PET-CT assessments of abdominal aortic aneurysms.

Analysing the entire abdominal aorta as a single region would potentially dilute and obscure differences between aneurysmal and non-aneurysmal regions. We therefore divided the aorta into three anatomically defined regions that are easily identifiable on a CT angiogram and can be easily replicated. We also used the thoracic aorta as a non-aneurysmal control segment of aorta. We appreciate that thoracic aortic disease may have a different pathophysiology to abdominal aortic aneurysm disease, and there may be differences in microcalcification activity and radiotracer uptake. However, since the study question here was the method of PET quantification, we feel that using the thoracic aorta as a control is a valid reference comparison.

We have sought to address the problem of signal spillage from the physiological uptake of sodium [^18^F]fluoride within vertebrae. Previous methods involved manually excluding obvious areas of activity spill-over from the vertebrae, and we applied this method when calculating the TBR values. Akerele et al. [14] have previously described other methods to correct for this problem including iterative reconstructions which incorporate a specific background correction that adjusts for this source of error. This is labour-intensive and currently there are no software packages to implement this technique. The PET activity spill-over takes place over a range of continuous values and its complete exclusion is not technically feasible. Our thresholding technique corrects for the abnormally high signal, but higher overall values of AMA_mean_ can still occur due to activity spill-over below the region’s set threshold. Despite this, we feel that this remains one of the more effective methods available to correct for the spill-over effect from intense vertebral sodium [^18^F]fluoride uptake because of its rapidity and simplicity as well as the improvement in comparative values with TBR_mean_.

The obtained AMA_mean_ value is dependent on a calculation involving the region’s cumulative SUV, region volume, region threshold and background SUVs. Disparities between different image analysts could potentially have an impact on the measured uptake values. However, the intra-observer and inter-observer repeatabilities were found to be very good if not excellent, especially after application of techniques to make the assessments more robust. Scan-rescan reproducibility has not been assessed within this method; however, it has already been shown to be very good in the thoracic aorta [6]. The dependence on the region’s volume could result in larger aneurysm diameters reducing the region’s AMA value. When developing this method, we considered using the length of the volume of interest rather than its volume, but the values obtained were not comparable to TBR values.

Forsythe et al. [5] used the “most diseased segment” TBR_max_ approach to measure sodium [^18^F]fluoride uptake in abdominal aortic aneurysms. These values demonstrated higher signal for aneurysmal segments compared to non-aneurysmal segments. This is a well-established approach that has previously been used to quantify [^18^F]fluorodeoxyglucose uptake in aortic and carotid atheroma and sodium [^18^F]fluoride uptake in the aortic valve [15–17]. The AMA_mean_ method described here is similar to the TBR_mean_ value: it calculates the average activity across a region of interest but it does not aim to replicate the “most diseased segment” approach which is dependent on a single voxel value across a region of interest. This explains the lower values in the aneurysmal segments in the present study. AMA_max_ would be more similar to this method; however, it compares less well to the TBR_max_ across the region. The “most diseased segment” method is valuable when investigating conditions where regions of intense activity are more important than mean global activity. For example, this has been used as a measure of atherosclerotic disease activity and the risk of plaque rupture in coronary artery disease [10, 18]. It is unknown whether aneurysm rupture or expansion are dependent on the most intensely active degenerative region in the aneurysm (which would correspond to the “most diseased segment”) or whether these events may be better reflected through a global average measure of the burden of vascular degeneration within the whole vessel (AMA_mean_).

It is important to highlight some limitations to our study. Whilst we have introduced enhancements in our technique to deal with the spill-over effect from physiological vertebral uptake, this remains a source of error and it is unclear whether our method adequately corrects for this. Since it is not possible with the current technology to have zero signal spillage with this radiotracer, calculating a true mean error is challenging. Some more sophisticated spill-over correction methods could be performed in the future, but they may require availability of dynamic imaging. Our study population consisted of patients with abdominal aortic aneurysms and we have not assessed our technique in a truly healthy population or other diseased states. There is also some dependence on the total volume of interest using our method. One potential way to improve direct assessment of the aortic aneurysm would be to have a hollow cylindrical volume of interest and thereby consider only the vessel wall itself. However, this incorporated increased complexity, took greater analysis time and performed poorly between different observers. We have sought to quantify sodium [^18^F]fluoride uptake in abdominal aortic aneurysms. This radiotracer has not been validated for clinical use and future studies are needed to determine if this AMA method can serve as a biomarker for aortic disease. We recognise that nuclear medicine departments may not routinely perform a contrast-enhanced CT acquisition. Hypothetically, a recent contrast-enhanced CT scan could be co-registered to a PET acquisition scan to allow more accurate determination of the aortic regions, and arterial landmarks.

## Conclusion

This study demonstrates a method of quantifying sodium [^18^F]fluoride uptake across the abdominal aorta. This method is quicker, less labour-intensive and simpler to apply. It demonstrated good intra-observer and inter-observer repeatabilities and provides measures of PET activity that are comparable to established methods.

## Supplementary Information

**Additional file1.** **Figure S1:** Radiotracer uptake in different regions of the aorta, Maximum tissue-to-background ratio (red) and maximum aortic microcalcification activity (blue) in the regions of the abdominal aorta and the thoracic aorta. AMA, aortic microcalcification activity; max, maximum; TBR, tissue-to-background ratio. * = p < 0.05, ** = p < 0.01, *** = p < 0.001, **** = p < 0.0001. **Figure S2:** Scatter plots of the different values quantifying sodium [18F]fluoride in the abdominal aorta, TBR_max_ and AMA_max_ values (a), TBR_mean_ and AMA (b), one observer performing the same AMA method twice (c), two observers performing the same AMA method (d). AMA, aortic microcalcification activity; max, maximum; R, Pearson’s correlation coefficient; TBR, tissue-to-background ratio.

## Data Availability

The data that support the findings of this study are available from the corresponding author upon reasonable request.

## Abbreviations

PET: Positron emission tomography
CT: Computed tomography
AMA: Aortic microcalcification activity
SUV: Standard uptake value
TBR: Tissue-to-background ratio
