# Supplementary material for: Quantifying sodium [18F]fluoride uptake in abdominal aortic aneurysms
Source: EJNMMI Res. 2022 Jun 6;12:33. doi: 10.1186/s13550-022-00904-z (PMC9170850; doi:10.1186/s13550-022-00904-z)
Supplement: Supplementary file 1 — Additional file1. Figure S1: Radiotracer uptake in different regions of the aorta, Maximum tissue-to-background ratio (red) and maximum aortic microcalcification activity (blue) in the regions of the abdominal aorta and the thoracic aorta. AMA, aortic microcalcification activity; max, maximum; TBR, tissue-to-background ratio. * = p < 0.05, ** = p < 0.01, *** = p < 0.001, **** = p < 0.0001. Figure S2: Scatter plots of the different values quantifying sodium [18F]fluoride in the abdominal aorta, TBRmax and AMAmax values (a), TBRmean and AMA (b), one observer performing the same AMA method twice (c), two observers performing the same AMA method (d). AMA, aortic microcalcification activity; max, maximum; R, Pearson’s correlation coefficient; TBR, tissue-to-background ratio. [file 13550_2022_904_MOESM1_ESM.pdf]

Quantifying sodium [^{18}F]fluoride uptake in abdominal aortic aneurysms

European Journal of Nuclear Medicine and Molecular Imaging

Samuel Debono[a], Jennifer Nash[a], Alexander J. Fletcher[a], Maaz B. J. Syed[a], Scott I. Semple[a], Edwin J. R. van Beek[a,b], Alison Fletcher[b], Sebastien Cadet[c], Michelle C Williams[a], Damini Dey[c], Piotr J. Slomka[c], Rachael O. Forsythe[a], Marc R. Dweck[a], David E. Newby[a]

Author Affiliations

[a] The University of Edinburgh Centre for Cardiovascular Science, University of Edinburgh, Edinburgh, United Kingdom

[b] Edinburgh Imaging Facility, Queen's Medical Research Institute, University of Edinburgh, Edinburgh, United Kingdom

[c] Departments of Medicine (Division of Artificial Intelligence) and Biomedical imaging Research Institute, Cedars-Sinai Medical Centre, Los Angeles

Corresponding author

Dr Samuel Debono
Chancellor's Building,
University of Edinburgh
49 Little France Crescent
Edinburgh, United Kingdom, EH16 4SB
E-mail: samuel.debono@ed.ac.uk

Supplementary Figure 1

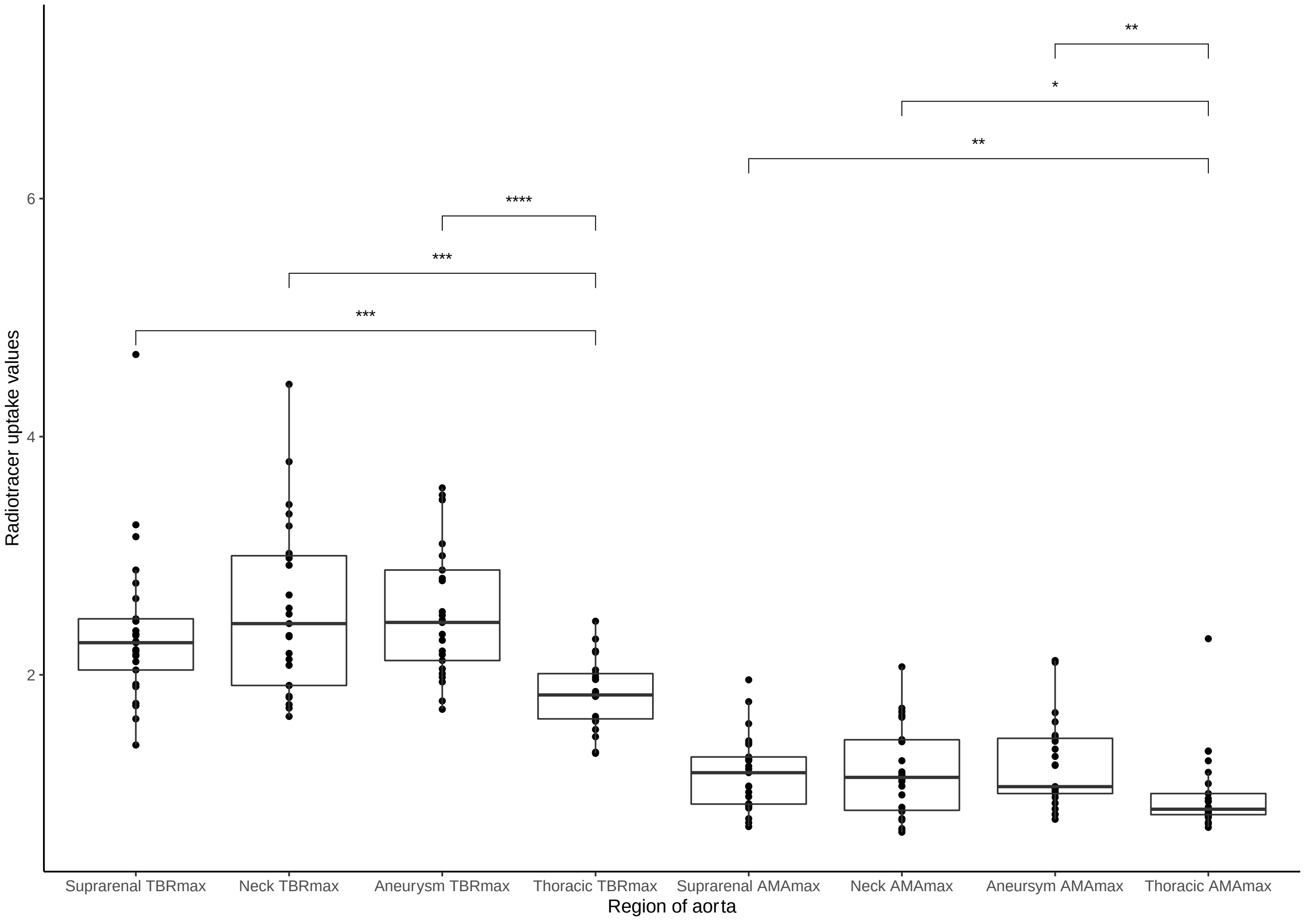

Supplementary Figure 1. Radiotracer uptake in different regions of the aorta. Maximum tissue to background ratio (red) and maximum aortic microcalcification activity (blue) in the regions of the abdominal aorta and the thoracic aorta. AMA, aortic microcalcification activity; max, maximum; TBR, tissue to background ratio. * = $p < 0.05$, ** = $p < 0.01$, *** = $p < 0.001$, **** = $p < 0.0001$.

Supplementary Figure 2

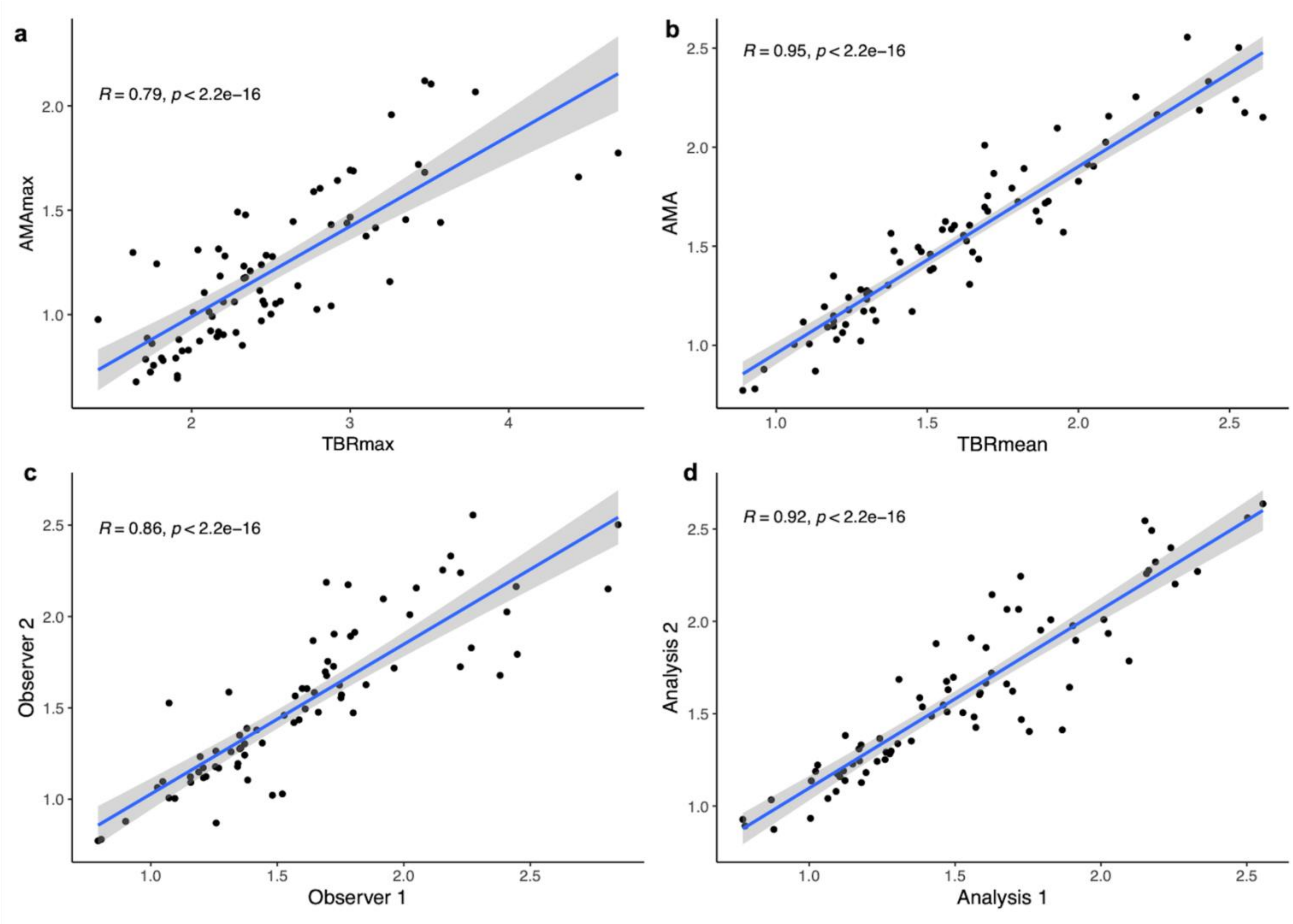

Supplementary Figure 2. Scatter plots of the different values quantifying sodium [^{18}F]fluoride in the abdominal aorta. TBR_{max} and AMA_{max} values (a), TBR_{mean} and AMA (b), one observer performing the same AMA method twice (c), two observers performing the same AMA method (d). AMA, aortic microcalcification activity; max, maximum; R, Pearson's correlation coefficient; TBR, tissue to background ratio.
